# Sub-PPB Detection with Gas-Phase Multiphoton Electron Extraction Spectroscopy under Ambient Conditions

**DOI:** 10.3390/s24072040

**Published:** 2024-03-22

**Authors:** Tikhon Filippov, Elena Vervitski, Hila Kofler, Lea Birkan, Shaked Levy, Shay Zimmerman, Valery Bulatov, Israel Schechter, Roman Schuetz

**Affiliations:** Schulich Faculty of Chemistry, Technion-Israel Institute of Technology, Haifa 32000, Israel

**Keywords:** gas-phase MEES, multiphoton electron extraction spectroscopy, trace gas analysis, electronic nose (e-nose), gas trace detection, environmental monitoring, security screening, explosives detection, breathomics

## Abstract

Multiphoton electron extraction spectroscopy (MEES) is an advanced analytical technique that has demonstrated exceptional sensitivity and specificity for detecting molecular traces on solid and liquid surfaces. Building upon the solid-state MEES foundations, this study introduces the first application of MEES in the gas phase (gas-phase MEES), specifically designed for quantitative detection of gas traces at sub-part per billion (sub-PPB) concentrations under ambient atmospheric conditions. Our experimental setup utilizes resonant multiphoton ionization processes using ns laser pulses under a high electrical field. The generated photoelectron charges are recorded as a function of the laser’s wavelength. This research showcases the high sensitivity of gas-phase MEES, achieving high spectral resolution with resonant peak widths less than 0.02 nm FWHM. We present results from quantitative analysis of benzene and aniline, two industrially and environmentally significant compounds, demonstrating linear responses in the sub-PPM and sub-PPB ranges. The enhanced sensitivity and resolution of gas-phase MEES offer a powerful approach to trace gas analysis, with potential applications in environmental monitoring, industrial safety, security screening, and medical diagnostics. This study confirms the advantages of gas-phase MEES over many traditional optical spectroscopic methods and demonstrates its potential in direct gas-trace sensing in ambient atmosphere.

## 1. Introduction

The precise detection, measurement, and monitoring of trace concentrations of substances in gaseous environments are critical for a multitude of applications that impact public safety, environmental health, and industrial processes. These applications range from enhancing security through identification of explosive materials to preserving ecological integrity by tracking atmospheric pollutants. Additionally, ensuring industrial safety by detecting hazardous gas leaks and advancing medical diagnostics through breath analysis are among the other pivotal uses of trace gas detection.

Putting the focus on medical diagnostics as an important application example, recent advances in breath analysis have shown promising results for non-invasive disease diagnosis and monitoring, leveraging the detection of volatile organic compounds (VOCs) as biomarkers for various conditions, including respiratory diseases, cancer, and metabolic disorders [1,2,3,4]. This burgeoning field, often referred to as ‘breathomics’, employs sophisticated analytical techniques such as gas chromatography-mass spectrometry (GC-MS) and electronic noses (e-noses) [5,6]. Despite its potential, breath analysis faces significant challenges. The complexity of breath composition, the influence of external factors on VOC concentrations, and the need for standardization in sampling and analysis are among the primary hurdles [7]. Moreover, the low concentrations of disease-specific biomarkers present in breath necessitate highly sensitive and selective detection methods to distinguish them in situ under ambient conditions from the myriad of other compounds present in breath [8]. These limitations underscore the need for further research to refine analytical methods and establish robust, clinically relevant breath biomarkers [9].

Despite the importance of these tasks, contemporary direct and on-line analytical techniques often fall short when it comes to accurately detecting and quantifying trace concentrations in the sub-parts per billion (sub-ppb) range under ambient conditions. This is especially true when high selectivity is needed in addition to sensitivity. The limitations of current technologies typically necessitate either preconcentration of analytes or establishment of controlled environments, which may involve high vacuum conditions and regulated temperatures, to achieve the desired measurement precision [10,11,12]. Some laboratory analytical instruments allow for extremely low limits of detection for some contaminants in gas samples [13]. In most cases, achieving high sensitivity requires a preconcentration step, which compromises the response time and is only effective for some specific chemical groups. Although fast chromatographic instruments are available, good separation of mixtures requires considerable chromatographic retention times. Regular optical spectroscopy technologies, including laser-based systems, provide real on-line detection. However, their specificity and their ability to handle mixtures are only of moderate strength, and their limits of detection are above the ppb range. The sensitivity of laser induced fluorescence-based detectors is much lower, but these detectors are limited to fluorescing compounds. Therefore, there is a need for new detection technologies which have the potential for sensitive on-line measurements, and which provide rich analytical information that can result is good identifiability and handling of mixtures.

Multiphoton ionization (MPI) is a process in which several photons from a short laser pulse are simultaneously absorbed by a molecule, resulting in ionization. It has been utilized in analytical chemistry for decades. The process has mainly been used in mass spectrometry (MS), during which molecular masses and their fragments are analyzed and identified [14,15,16]. In most MPI-MS configurations, a single laser wavelength is utilized; however, resonance-enhanced ionization and two-laser ionization have also been reported. In these setups, ionization takes place in a single small spot where the number of analyte molecules is limited. This limitation is related to the need to manipulate the generated ions in mass spectrometric detectors. Another limitation in the above mass spectrometric methods is the required high vacuum conditions, needed for providing long mean free pass of the ions. A partial solution to these drawbacks has been suggested: using an unfocused laser beam and monitoring the time-dependent mirror charges [17]. In this method, the laser beam generates ions along its pass, and they move towards a nearby negative electrode. The distance to the electrode is short (ca. 1 cm), so only a moderate vacuum (ca. 10^−5^ torr) is needed to allow for collision free mobility. Such a short time-of-flight does not allow for effective analysis; thus, detection is based on measuring the time dependent mirror charges induced in the electrodes during their flight. Instead of identification based on a single value of the time-of-flight (or arrival location, in alternative systems), numerous data were collected for each ion during its flight. This approach partially compensated for the errors due to the short pass. This method has allowed for the ionization of a much larger number of molecules; however, it severely compromises mass identification, and only simple mixtures could be properly resolved.

Multiphoton ionization under ambient conditions (atmospheric pressure, temperature, and composition), performed at a single laser wavelength, has also been applied for analysis of solid and liquid surfaces [18,19,20]. The results indicated high sensitivity to a variety of molecules, but no identification was possible under such conditions.

An emerging solution to these challenges is multiphoton electron extraction spectroscopy (MEES), a new analytical method that was suggested for direct analysis of solids and liquids under ambient conditions. MEES utilizes short ultraviolet (UV) laser pulses to excite and ionize samples in a multiphoton process. Unlike in MS methods, photoelectrons are recorded, which is a much simpler, more accurate, and lower cost process as compared to the recording of ions. This also eliminates the need for vacuum conditions. The resulting time-resolved current is integrated, providing the total emitted photocharge. Detection is based on the spectrum of photocharges as a function of the laser wavelength. In this kind of spectroscopy, numerous sharp peaks are observed. They are related to resonant transitions between the molecular electronic and vibronic energy levels of the analyte. A peak is generated when the energy of any combination of photons is equal to the transition of the molecule to any of its excited states. Therefore, the number of peaks in this spectroscopy is much larger than in absorption or emission spectroscopies. Very high energy levels of the molecule can also be revealed. The density of peaks (number of peaks per a given wavelength range) in MEES is much larger than in all single photon spectroscopies. This results in more analytical information, which is beneficial for identification and for handling of mixtures.

The superb sensitivity of MEES is attributed to the high ionization efficiency at resonant wavelengths, to the high (almost 100%) charge collection efficiency under the high electrical field applied, and to the fact that electrical charges are measured over a low background, rather than changes in photon flux. High amplification at very low noise level is easily obtained in electronics, and the system’s detection-limiting noise level is primarily determined by the quality of the electronic devices involved.

So far, MEES has only been developed and characterized for the analysis of surfaces [21,22,23,24]. Extraordinary sensitivity (in the fmole range) has been reported for direct analysis under ambient conditions. Besides its sensitivity, what sets surface-MEES apart is the high density of sharp peaks, which allows compound fingerprinting and excellent identifiability [25]. The analytical performance of MEES has been compared to that of most common optical spectroscopies and was found to be superior in terms of both sensitivity and analytical information [25].

In this paper, we report a new experimental setup, which was designed for applying MEES to the gas phase. This is the first report on using gas-phase MEES technology for sensing trace organic compounds. Our findings highlight the potential of gas-phase MEES to transcend the capabilities of existing analytical methods [26,27,28,29,30], offering a versatile, real-time solution for environmental monitoring, homeland security, medical diagnostics, food safety, quality control, and beyond.

The transition from solid MEES to gas-phase MEES necessitated various experimental adjustments, outlined below. The gas flow eliminates the necessity to refresh the irradiated spot. However, in gas-phase MEES, time-resolved signals are influenced by positively charged species. In contrast, in solid MEES, these species remain bound to the surface and are promptly neutralized by electrons from the grounded electrode.

In this paper, gas-phase MEES potential is showcased through various demonstrations. Signal linearity across a broad concentration range is illustrated using volatile compounds, and sensitivity in the sub-ppb range is demonstrated with low-volatile compounds. Gas-phase MEES proves to be an exceptionally sensitive and informative analytical method, surpassing the capabilities of most optical spectroscopies.

## 2. Materials and Methods

### 2.1. Experimental Setup

**Figure 1:**
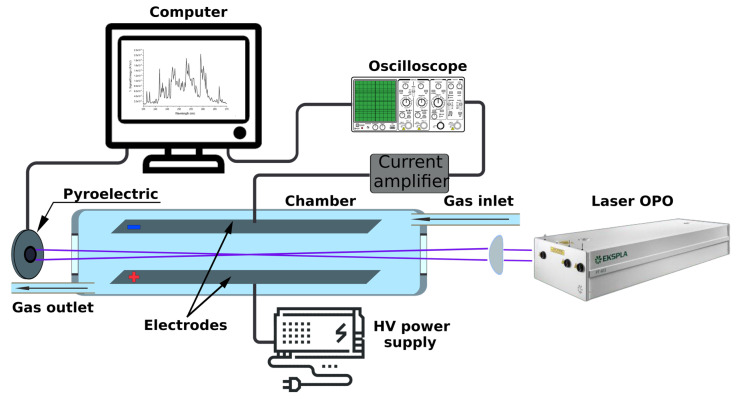
The experimental setup with gas flow chamber system.

Our home-made gas-phase multiphoton electron extraction spectroscopy (gas-MEES) system was meticulously assembled to facilitate the measurement of molecular traces in gases at ambient conditions. Figure 1 presents a block diagram of the experimental setup. In a faraday cage flow chamber, two stainless steel electrodes 22 cm long and 2.5 cm wide were positioned 1–2 cm apart (the distance is variable). A high voltage supply (PS325, Stanford Research Systems) was connected to one of the electrodes to provide an up to 5 kV/cm electric field. The second electrode was connected to a current amplifier (Variable Gain Low Noise Current Amplifier DLPCA-200, FEMTO Messtechnik GmbH, Berlin, Germany) and an oscilloscope (Picoscope 3000 series, Pico Technology, Cambridgeshire, UK) to form the time-resolved detecting system. The picoscope was triggered by the laser’s internal trigger pulse. Nitrogen gas of high-grade purity (99.999%) was used as a carrier gas, and air was provided by the institute’s compressor to the lab.

The light source was a solid-state OPO pulsed laser (NT 342/3/UVE EKSPLA, EKSPLA, Vilnius, Lithuania). The laser offered a wide range of wavelengths in the UV range, from 192–410 nm with 0.02 nm step resolution. The repetition rate was 10 Hz, and the pulse duration was about 2 ns. The laser pulse energy was between 0.2 mJ and 2.5 mJ. The intensity of the laser at each wavelength was monitored by a pyroelectric sensor (Ophir Laserstar PE-10-sh-v2, Ophir Optronics Solutions Ltd., Jerusalem, Israel). The beam was introduced to the chamber through a 1-inch quartz window. The core configuration consisted of two electrodes with a 2 kV/cm electrical field applied between them. The laser beam was mildly focused by a lens (*f* = 30 cm) in the center between the electrodes. The mild focusing over such a long focal length was carefully selected and tested in order to avoid plasma creation in the gas phase between the electrodes, while still achieving high EM field densities along the beam.

### 2.2. Gas Flow Chamber System

A flow design for the chamber was chosen due to several advantages over static design. First, it simulated an on-line sensor setup. The flow design also ensured uniform exposure of the gas to the laser pulses, mitigating the effects of diffusion and adsorption/desorption processes, and reducing the influence of potential photochemical reactions. This setup significantly reduced the risk of contamination, providing a controlled and consistent experimental environment.

### 2.3. Representing Analytes

Benzene and aniline were chosen as representing analytes due to their significance in organic chemistry and industry, and their environmental impacts. Benzene, a typical aromatic hydrocarbon, was used to assess the system’s performance in the sub-ppm and sub-ppb concentration ranges in both pure nitrogen and air. Additionally, aniline, an aromatic amine, also significant due to a wide application range such as in pharmaceuticals, dyes, and organic synthesis, was employed for studying the sub-ppb range. The accurate detection and quantification of these VOCs are critical for health and safety, compliance with regulatory standards, and environmental monitoring.

### 2.4. Measurement and Data Acquisition

Upon photo-ionization, the generated molecular ions and photoelectrons were collected by the electrodes. The resultant time-dependent current was then amplified using a current amplifier and recorded with a pico-scope. The pico-scope was synchronized with the laser pulses by adjusting its trigger.

The setup operation, data acquisition, and processing tasks were automated through a custom-built software suite developed using VB.NET code. After recording the waveforms of the photocurrent as a function of time, these data were integrated to obtain the total photo charge as a function of laser wavelength. Laser intensity (from the power meter) was also recorded, so that normalization of the total photo charge to the beam energy could be carried out.

### 2.5. Sample Preparation and Dilution

Two distinct well-established methods were employed for the preparation and dilution of the specimen gases to achieve the desired concentration ranges:

1. Permeation Tube Method: PTFE permeation tubes were used in experiments with benzene. The permeation tube was placed in a glass bubbler before the chamber inlet. A stable flow (nanograms-per-minute) of analyte vapor was emitted through the tube wall at constant temperature [31]. The emission rate of the tube was determined by measuring the rate of weight loss [31] over a long-term period. The temperature of the permeation tube was kept constant to maintain a constant output, while the carrier gas flow rate was adjusted to achieve the required concentrations. In order to cover both concentration ranges, sub-ppm and sup-ppb, while keeping the carrier gas flows economically moderate (nitrogen up to 3 L/min and air up to 26 L/min), different sizes of permeation tubes were used (big and small, respectively).
2. Syringe Pump Method [32]: This method was utilized for aniline at sub-ppb concentration ranges, and was characterized by its low efficiency to penetrate the permeation tubes. The syringe pump (Standard Infuse/Withdraw Pump 11 Elite Programmable Syringe Pump Harvard Apparatus, Holliston, MA, USA) with a micro syringe (2 µL Syringe Model 7102, Knurled Hub, Hamilton Company, Bonaduz, GR, Switzerland) delivered different precise dilutions of aniline in ethanol, with the gas flow rate held constant to attain the ultra-low concentrations needed for our measurements.

## 3. Results and Discussion

In MEES, the normal composition of ambient air does not interfere with many organic and inorganic analytes, simply because the ionization potential of the formers is higher. This means that more photons are needed for their ionization, and their multiphoton ionization cross section is much lower. Consequently, their ionization probability is also much lower [16].

**Figure 2:**
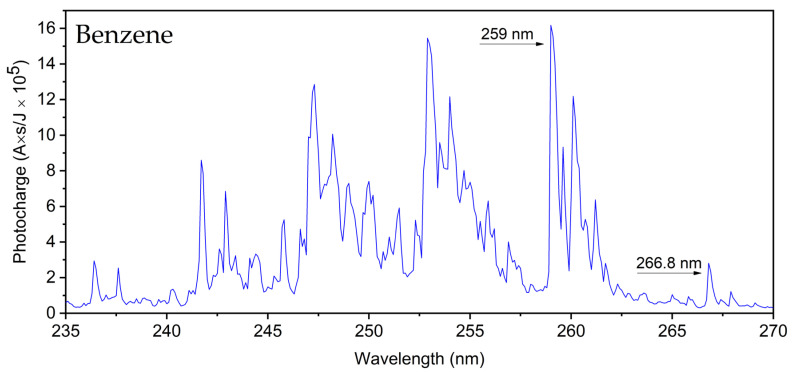
A representative benzene spectrum in the nitrogen environment at 150 ppb. The peaks used for the calibration are indicated by the dashed arrows: 266.8 nm and 259 nm for the high (sub-ppm) and ultralow (sub-ppb) concentrations, respectively.

In this study, we employed a newly constructed gas-MEES setup to measure molecular traces in gases under atmospheric conditions with sub-ppb sensitivity. Our experimental design involved the use of a flow chamber to ensure uniform gas exposure and minimize contamination, paired with a pulsed laser for the ionization of gas molecules between two electrodes (Figure 1). The resultant current waveforms were recorded over time, and the integrals of these peaks were plotted to generate a photocharge spectrum as a function of the laser wavelength (Figure 2). Notably, in the presence of atmospheric oxygen, the photoelectron pulse was considerably lower compared to that in the nitrogen environment. However, with an increase of the voltage between the electrodes, the photoelectron pulse current rose. Increased electrode voltages can lead to saturation in analyte detection and to nonlinear calibration characteristics of the analyte concentrations. It is therefore important to adjust the instrumental parameters appropriately according to the specific range of sensitivity in order to ensure best performance. For the purposes of this study, we focused exclusively on the integral of the sharp electron peak.

Figure 2 presents the MEES spectrum of Benzene measured at 0.1 nm resolution. This low resolution was used in order to present the full spectrum in a wide range. However, the laser’s best resolution is 0.02 nm, which was actually used in our quantitative measurements. The high resolution spectra allow for much better analyte identification. These peaks appear in several groups. Each of them is related to an electronic transition, while the smaller peaks within groups are related to the corresponding vibronic states. Some of the measured peaks are those observed in the absorption spectrum of benzene gas, but multiphoton absorption samples have many more energy levels, especially the higher ones. Spectral assignment of the MEES peaks requires calculation of all energy levels of the relevant molecules, and this had not yet been performed for most organic compounds.

Accordingly, benzene calibration was performed on the peaks at 266.80 nm (4.647 eV) and 259.00 nm (4.787 eV), which correspond to the $B_{0}^{0}$, $6_{1}^{0}$ and $A_{0}^{0}$, $6_{0}^{1}$ bands of the $B_{2u}\leftarrow A_{1g}$ electronic transition [33]. These were used for the high (sub-ppm) and ultralow (sub-ppb) concentrations, respectively. Similarly, aniline calibration was based on the integrated signals in the range of 285.82 nm (4.338 eV), which corresponded to 1$2_{0}^{1}$ transition ($B_{2u}\leftarrow A_{1g}$) [34] and was used for the ultrahigh sub-ppb region.

### 3.1. Sub-PPM Range

**Figure 3:**
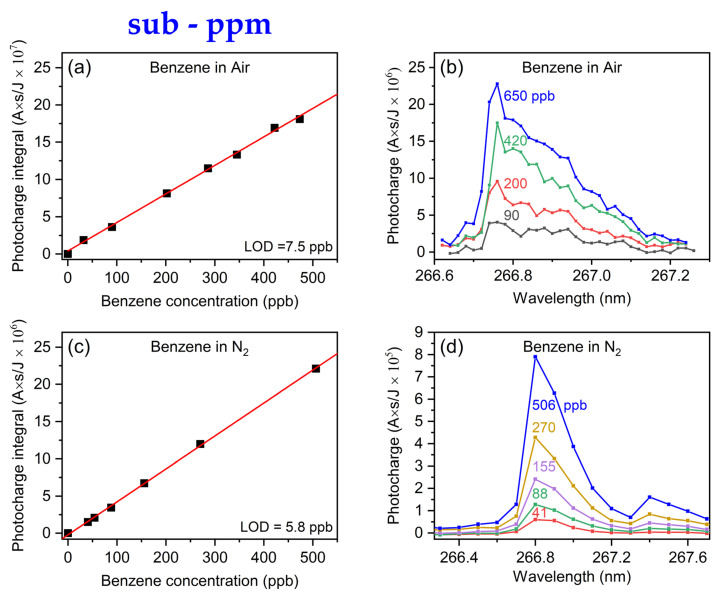
(**a**,**c**): Calibration curves for the sub-ppm range of benzene in air and in nitrogen environments, respectively. (**b**,**d**) The corresponding spectral region of integration at selected concentrations.

We initially focused on the sub-ppm range using benzene, a compound of high vapor pressure (74.6 torr), to validate the linear response of the gas-MEES system in ambient air and in nitrogen. Utilizing a permeation tube, we were able to regulate benzene concentrations within the 0–650 ppb range by varying the flow rate of the carrier gas. The results are shown in Figure 3. The results demonstrate a clear linear relationship between the photocharges and benzene concentration, confirming the system’s capability for quantitative detection in this region in air and in nitrogen. The currently determined 95% based limits of detection (LODs) were 7.5 ppb and 5.8 ppb, respectively.

These results can be used as a calibration procedure for determining benzene in air using gas-phase MEES. It does not require any calibration standards, since it is directly based on accurate sample preparation procedures (as described in the experimental section).

Using these measurements, a linear calibration was achieved up to 650 ppb. However, at higher concentrations, saturation occurred, potentially due to the generation of space charges. To extend the linear dynamic range, a straightforward approach is to reduce either the laser intensity or the intensity of the applied electrical field.

### 3.2. Sub-PPB Range

**Figure 4:**
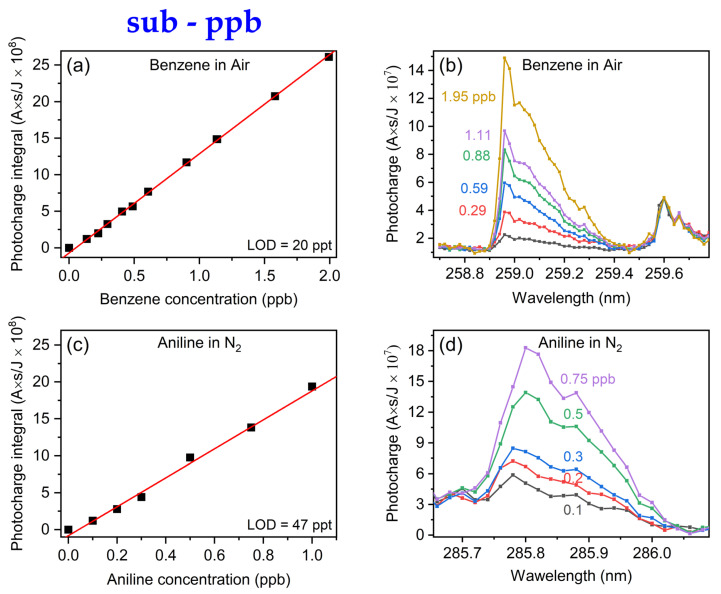
(**a**,**c**): Linear calibration curves for sub-ppb range of benzene in air and in aniline in nitrogen atmospheres, respectively. (**b**,**d**) show the corresponding spectral region of integration at the selected concentrations.

In order to explore the sub-ppb sensitivity range, we extended our analysis to aniline, a compound with a lower vapor pressure (0.6 torr). However, in contrast to benzene, aniline showed no mass loss through the walls of the permeation tubes, and subsequently, no spectrum could be obtained using this approach. We therefore applied the syringe pump method for aniline. By employing a syringe pump and varying dilutions in ethanol, we meticulously controlled the aniline concentrations below 1 ppb. The resulted calibration curves for benzene and aniline in air and in nitrogen environments are shown in Figure 4a,c. The results once again exhibited a linear response, extending the system’s demonstrated sensitivity to the sub-ppb level. The 95% based LODs for the presented calibration curves of benzene in air and aniline in nitrogen are 20 ppt and 47 ppt, respectively.

The corresponding gas-MEES spectra are also shown in Figure 4b,c. The rich MEES spectral features, together with the high spectral resolution, can be used for material fingerprinting and identification of the analyte. However, this feature and handling identification of an analyte in a mixture have not been investigated in this paper.

In principle, the results in air should be very similar to those obtained in nitrogen. However, there are some differences: electrons moving in air are likely to be caught by oxygen molecules, forming O_2_^−^. These heavy species travel much slower towards the electrode. Charge transfer may also take place in the many collisions. Therefore, the probability of the electron to reach the wall instead of the electrode is higher, which results in somewhat lower signals and LODs.

The time needed for acquiring a full MEES spectrum depends mainly on the laser repletion rate, the spectral resolution, the spectral range, and the number of pulses per wavelength. A typical spectrum at 10 Hz, 0.1 nm resolution, 1 nm range and eight pulses per wavelength takes ca. 10 s. The time needed for quantification at a single peak is much shorter.

MEES could be susceptible to certain persistent air impurities, which the baseline correction algorithm addresses. In this respect, it is essential to note that MEES provides a very detailed high-resolution spectrum of the analyte, allowing the selection of a wavelength with no interference.

The capability to quantify molecular traces at sub-ppb concentrations under ambient conditions in air or nitrogen atmospheres, without the need for preconcentration or controlled environments, has been demonstrated. However, we have not explored even lower concentrations simply because we could not produce such low reliable control concentrations in our lab. Nevertheless, we see potential for sensitivity detection down to ppt concentrations.

## 4. Conclusions

The development and implementation of a gas-phase multiphoton electron extraction spectroscopy apparatus has been achieved. This first study demonstrates its utility for the quantitative detection of volatile and low-volatile organic compounds in ambient air at both sub-ppm and sub-ppb concentration.

The results indicate that gas-phase MEES surpasses the detection capabilities of many existing on-line analytical spectroscopic methods in terms of sensitivity and spectral information. The ability to detect benzene and aniline with such precision—without the necessity of analyte preconcentration, chromatographic separation, or specialized environmental controls—implies excellent potential of this technology in trace gas monitoring.

The heightened sensitivity of MEES is credited to its reliance on electron detection rather than photons, leading to an improved signal-to-noise ratio. The detection-limiting noise level of the system is primarily determined by the quality of the electronic devices involved and can thus enhanced in line with investment.

MEES outperforms other optical spectroscopies in fingerprinting due to its ability to sample a more extensive range of molecular electronic and vibronic energy levels. While compiling a database of MEES spectra can enhance analytical performance, it may not match up to the capabilities of mass spectrometric devices.

Considering the pressing need for advanced detection methods capable of monitoring trace levels of hazardous substances, gas-MEES shows considerable potential for applications across various sectors. These include environmental protection, where precise monitoring of air pollutants is critical; industrial safety, for the detection of gas leaks and ensuring compliance with health regulations and security, and for identifying explosive or harmful chemical agents; and the development of breath analysis techniques in the medical field for disease diagnosis and monitoring.

Gas-phase MEES is not devoid of limitations, and many remain unidentified. These may encompass potential matrix effects yet to be discovered. The baseline correction algorithm effectively mitigates constant gas impurities. However, challenges may arise with variable gas impurities, although in many instances, this can be resolved by wavelength switching. High concentrations of organic aerosols can potentially lead to inaccurate readings.

Future work is needed to expand the range of detectable compounds, refine the apparatus to achieve even greater sensitivity, and explore portable configurations for in-field measurements. In the current work, the temperature and humidity were not varied and investigation of the effect of these parameters on the spectra is still needed. Handling of analyte mixtures is also an important challenge. However, the abundancy of sharp peaks indicates a high potential for mixture analysis. In condensed-phase MEES, although the peaks are not that sharp, simultaneous analysis of simple mixtures has been demonstrated. Thus, we hope that the performance in gas-phase MEES will be better. This is a new measurement technology; therefore, compiling libraries of MEES spectra of a variety of compounds is also needed. The successful application of surface MEES in detecting many compounds indicates that the methods involved can be extended to the gas-phase. Further enhancement of sensitivity is in the planning stages. This can be accomplished through the optimization of laser power, applied voltage, and the distance between electrodes. A significant additional improvement can be achieved by increasing the number of probed molecules, achieved by expanding the laser beam and elongating the measurement chamber.

## Data Availability

Data is contained within the manuscript.
